# Consequences of Neglect: Analysis of the Sub-Saharan African Snake Antivenom Market and the Global Context

**DOI:** 10.1371/journal.pntd.0001670

**Published:** 2012-06-05

**Authors:** Nicholas I. Brown

**Affiliations:** 1 Masters of Bioscience Enterprise Program, Institute of Biotechnology, The University of Cambridge, Cambridge, United Kingdom; 2 Australian Venom Research Unit, University of Melbourne, Melbourne, Australia; Liverpool School of Tropical Medicine, United Kingdom

## Abstract

**Background:**

The worldwide neglect of immunotherapeutic products for the treatment of snakebite has resulted in a critical paucity of effective, safe and affordable therapy in many Third World countries, particularly in Africa. Snakebite ranks high among the most neglected global health problems, with thousands of untreated victims dying or becoming permanently maimed in developing countries each year because of a lack of antivenom—a treatment that is widely available in most developed countries. This paper analyses the current status of antivenom production for sub-Saharan African countries and provides a snapshot of the global situation.

**Methods:**

A global survey of snake antivenom products was undertaken in 2007, involving 46 current and former antivenom manufacturers. Companies producing antivenom for use in sub-Saharan Africa were re-surveyed in 2010 and 2011.

**Results:**

The amount of antivenom manufactured for sub-Saharan Africa increased between 2007 and 2010/11, however output and procurement remained far below that required to treat the estimated 300,000–500,000 snakebite victims each year. Variable potency and inappropriate marketing of some antivenoms mean that the number of effective treatments available may be as low as 2.5% of projected needs. Five companies currently market antivenom for sale in Africa; three others have products in the final stages of development; and since 2007 one has ceased production indefinitely. Most current antivenom producers possess a willingness and capacity to raise output. However inconsistent market demand, unpredictable financial investment and inadequate quality control discourage further production and threaten the viability of the antivenom industry.

**Conclusion:**

Financial stimulus is urgently needed to identify and develop dependable sources of high-grade antivenoms, support current and emerging manufacturers, and capitalise on existing unutilised production capacity. Investing to ensure a consistent and sustainable marketplace for efficacious antivenom products will drive improvements in quality, output and availability, and save thousands of lives each year.

## Author Summary

Antivenom is the only specific treatment for systemic envenoming from snakebite, but remains unavailable to thousands of snakebite victims around the world. A cycle of inconsistent and low market demand, sub-optimal utilisation, rising costs and reduced output of antivenoms have resulted from long term under-investment in procurement and quality regulatory programs. This study provides a contemporary overview of the African antivenom market within the context of the global market. Globally, 35 companies sold at least 4 million vials of antivenom in 2007. Five companies had established African antivenom markets in 2010/11; three other institutions have antivenoms for Africa in development; and another ceased production indefinitely. Between 2007 and 2011, production of sub-Saharan African antivenoms rose from 227,400 to at least 377,500 vials, constituting ∼83,000 effective treatments for moderate envenomings. However, recent reports have identified that some products, which comprise up to 90% of the total antivenom supply in sub-Saharan Africa, may lack efficacy or specificity against relevant snake species. Despite this, revenues from antivenom marketed in sub-Saharan Africa increased from $6.6 million in 2007 to $10.3 million in 2010/11. The average cost of a stated effective treatment in 2010/11 was $124, and the price of antivenom is inversely proportional to the amount produced. Combined unutilised production capacity far exceeds the total projected antivenom needs for Africa.

## Introduction

Snakebite is a significant social and economic problem in many developing countries, however its victims rank among those most neglected by global health campaigns. Snakebite was recognised by the WHO as a Neglected Tropical Diseases in 2007, and antivenom – the only specific treatment for systemic envenoming - remains largely inaccessible to hundreds of thousands of snakebite victims around the world. Since its introduction and continued refinement throughout the twentieth century, antivenoms have saved countless lives [1]. Although readily available in wealthy countries and able to reduce mortality rates to less than 1% [1]–[3], sources of effective, safe and affordable antivenom in low-income countries, where the incidence of snakebite is greatest, are highly variable. Whilst good quality products do exist in some developing countries its procurement is often inadequate, leaving snakebite victims without access to proper treatment. Quantifying the gap between what is currently available and what is needed is a critical step towards developing effective solutions to this problem. This study provides a contemporary overview of global antivenom production, focusing particularly on the antivenom market in sub-Saharan Africa.

### 1. The rise and fall of antivenom

Since Edward Jenner's controversial inoculation of James Phipps with cowpox in 1796, immunotherapy has developed into a diverse industry [4]. Calmette's groundbreaking work with equine antiserum resulted in the first, unrefined antivenom in 1894. Pope's improvements to antivenom refinement in the 1930s were another major step forward in safety and potency of antivenom. Unfortunately, further advances since then have been limited. Despite snakebite being over-represented in morbidity and mortality tables [5], investment in this type of immunotherapy has not been characterised by the same level of publicity or resolve that has characterised vaccine production or monoclonal antibody research. This under-recognition of bites and stings as major medical and social problems, and snakebite's association with poverty, have contributed to the current antivenom crisis [6].

The introduction of antivenom to Africa in the 1950s heralded a decline in morbidity and mortality from snakebites that led to its widespread use and production. Sadly, over the last 30 years, production of this life-saving medication has been neglected by governments and non-government organisations, and abandoned by some manufacturers [7]. The 1970s and 1980s were characterised by a decline in the sale of antivenom in Africa due to growing neglect and prohibitive costs [8]. By 1998, it was estimated that fewer than 100,000 vials of antivenom were available across Africa, constituting less than 25% of the amount needed [9]. A number of recent publications state the availability of antivenom in Africa has reduced to <1% of what is needed, or “<20,000 vials reduced from ∼250,000 doses/year 25 years ago” [10]. The WHO has estimated that antivenom supply failure in Africa is imminent [11], which is further compounded by the presence of non-specific or fake products, inappropriate clinical use and poor community awareness of the benefits of antivenom [12]–[14].

## Methods

Data for this paper was collected from primary and secondary sources, including interviews, surveys, product inserts and literature searches. Market research surveys were sent to representatives from 46 known antivenom manufacturers in 2007. Previous, current and future producers for sub-Saharan African markets were again contacted in 2010 and 2011. One current and one future company did not respond in 2010/2011. Companies responded with information regarding the following:

- quantity and type of antivenom produced, including number of unsold vials;
- average number of snake antivenom vials required to successfully treat a moderately severe snake envenomation;
- countries where antivenom is marketed;
- wholesale cost of antivenom;
- existing spare production capacity;
- quality control and regulatory standards;
- profitability of antivenom products; and
- attitudes about the use, availability and sustainability of antivenom.

Calculations regarding the number of vials that constitute an “effective treatment” are based on company information and product inserts for an average, or moderately severe, envenomation. Independent testing of potency and proteomic analysis to validate the species of origin was outside the scope of this study, although verification was sought through literature reviews.

## Results

### 1. Epidemiological estimates of antivenom requirement

The global incidence of clinically significant snakebite has been calculated to be between 421,000 and 2.5 million annually [15], [16], with up to 500,000 occurring in Africa each year [10], [17], [18]. Inadequate record keeping and limited primary epidemiological studies makes accurate assessment difficult, and most authors concur that estimates of snakebite incidence under-represent the problem. Up to 20–70% of victims in some regions do not present to hospital because they are either unaware treatment is available, cannot afford it, or instead utilise ineffective traditional healing methods [19]–[21]. However a recent metaanalytical study of reported data concluded that probably 314,000 snakebites occur in Africa annually [22]. The rate of snakebite in sub-Saharan Africa varies between 150–250/100,000 population [23]–[25], with a peak incidence in some regions in Nigeria of 497/100,000 [26].

At least 20,000 deaths each year are attributed to snakebite in Africa [17], although this is also considered conservative. The recorded annual mortality in Nigeria, Senegal and Kenya is between 2–16/100,000 population, and across Africa the case fatality rates from untreated snakebite ranges from 4% to 24% [27]–[30]. The WHO estimates that 10% of envenomings results in serious, non-fatal sequelae, while other reports have stated that 12,000–14,000 amputations and other sequelae result from snakebites in Africa annually [19], [31], [32]. Other debilitating morbidities result from the neurotoxic, coagulopathic or necrotic components of different venoms, with clinical effects ranging from chronic ulceration, osteomyelitis, chronic renal failure, endocrine disorders, paralysis, stroke and blindness.

### 2. Current African antivenom market

#### a. Manufacturers (table 1)

**Table 1:** Recent and current sub-Saharan African antivenom manufacturer.

Company, country of origin	Antivenom type	Venoms used in immunisation	Countries antivenom is available
MicroPharm, United Kingdom	Mono; ovine; liquid (10 ml); intact IgG	*Echis ocellatus*, with cross specificity for other Echis species	Nigeria
Sanofi Pasteur, France	Poly; equine; F(ab)′2; lyophilised, (10 ml);	*Bitis gabonica*, *Bitis arietans*, *Echis leucogaster*, *E. ocellatus*, *Naja haje*, *N. melanoleuca*, *N. nigricollis*, *Dendroaspis polylepis*, *D. viridis*, *D. jamesoni*	West Africa, East Africa
South African Vaccine Producers, South Africa	Two×mono; one×poly; equine, F(ab)′2); lyophilised or liquid (10 ml)	*Dispholidus typus* (mono); *Echis ocellatus/carinatus* (mono); *Bitis arietans*, *B.gabonica*, *Haemachatus haemachatus*, *Dendroaspis angusticeps*, *D. jamesoni*, *D. polylepis*, *Naja nivea*, *N. melanoleuca*, *N. annulifera*, *N. mossambica* (poly)	South Africa, other African countries occasionally
VINS Bio, India	Poly; equine; liquid (10 ml) or lyophilised; >20–25 LD50	*Naja melanoleuca*, *N. nigricollis*, *N. haje*, *Dendroaspis polylepis*, *D. viridis*, *D. jamesoni*, *Bitis gabonica*, *B. arietans*, *Echis leucogaster*, *E. carinatus#; Daboia russelli#,*	Kenya, Nigeria, Ghana, Burkina Faso, Angola, Mozambique, Sudan
Bharat Serums and Vaccines, India	Poly; F(ab)′2 equine; lyophilised or liquid (10 ml);	*Bitis gabonica*, *B. arietans*, *B. nasicornis*, *Dendroaspis jamesonii*, *D. polylepis*, *D. angusticeps*, *Echis carinatus#*,*Naja nivea*, *N. nigricollis*, *N. haje*, *N. Melanoleuca*	Ghana, Nigeria, Kenya, Benin, Burkina Faso, Sudan
Serum Institute of India, India* (now discontinued)	Poly; equine; lyophilised (10 ml)	Bitis, Echis, Dendroaspis, *Daboia russelli#*	Ghana, Tanzania, Ethiopia, Kenya, Sudan
Instituto Bioclon, Mexico, N/A	Poly; equine; F(ab)′2; lyophilised	*Bitis arietans*, *B. gabonica*, *Echis ocellatus*, *E. Pyramidum, E. leucogaster*, *Naja naja*, *N. haje*, *N. nigricollis*, *N. pallida*, *Dendroaspis polylepis*, *D. Viridis*	West Africa; Post clinical trials; [44]
Instituto Clodomiro Picado, Costa Rica, N/A	Poly; equine; liquid; intact IgG	*Echis ocellatus*, *Bitis arietans*, *Naja nigricollis*	West Africa; Post clinical trials; [2]
Instituto Butantan, Brazil, N/A	Poly; Equine, F(ab)′2, liquid.	*Bitis arietans*, *B. nasicornis*, *B. rhinoceros*, *Naja malanoleuca*, *N. Mossambica*	Mozambique; in clinical trials [41]

(* manufacturer has now ceased antivenom production; # not an African species; poly = polyspecific; mono = monospecific; N/A = not yet available).

Between 2007 and 2010/11, six manufacturers sold antivenom for use in sub-Saharan Africa, although one has now ceased producing African antivenom indefinitely and another now only manufacturers antivenom to order after a lack of demand forced a temporarily hiatus of production in 2010. Three other institutions are developing antivenom against African snake species that have either recently been licensed or are in the final stages of development. Data on the planned output of antivenoms for Africa from these organisations is either not yet available or for experimental purposes only. Companies are based in the United Kingdom, France, South Africa, India, Mexico, Costa Rica and Brazil, with only one classified as “big pharma”.

A further three groups based in Egypt, Saudia Arabia and Iran produce antivenom against snake species found in West Asia and the Arabian peninsula, which may have efficacy against some North African snake species. Owing to their “off-label” nature for use against continental African snake species, these were not included in the final analysis. Another organisation, based in Colombia, appears to have suspended development of a pan-African antivenom after conducting preclinical work in 2003.

#### b. Antivenom output and capacity

Producers of sub-Saharan African antivenom had a combined annual output of at least 377,500 vials in 2010/2011, equating to approximately 83,000 complete treatments for moderate envenoming, based on manufacturers' recommended doses (table 2). By comparison, 227,400 vials of sub-Saharan African antivenom were marketed to African countries in 2007, providing just over 54,000 average treatments (table 3). In 2007, manufacturers reported a combined excess supply of more than 26,000 vials of unsold African antivenom. By 2010 no manufactured antivenom was unsold, however significant unutilised production capacity was reported by 5 of the 8 current producers, including two with manufacturing facilities and quality control procedures regulated by the European Medical Agency (EMEA). If utilised, this combined capacity could produce enough antivenom to treat 600,000 patients and save thousands of lives.

**Table 2:** 2007sub-Saharan African antivenom output and market.

Company	Vials produced per year	No. of vials (treatments) unsold in 2007	Wholesale cost per vial (US$)	Vials per average treatment	Complete treatments (average)	Cost of AVERAGE treatment	Value of African AV
A	10,000	5,000 (3,570)	$40	1–2 (Avg 1.4)	7,200	$56	$400,000
B	<2000	0	$135	3–4 (Avg 3.7)	<540	$500	$270,000
C	10,400	>1,000 (>125)	$80 (poly) $200 (mono)	6–10 (poly) 2 (mono)	1250 (poly) 200 (mono)	$640 (poly) $400 (mono)	$880,000
D	5,000	0	$18	4–9	770	$117	$90,000
E	100,000	>20,000 (>6,667)	$32	2–4	33,300	$96	$3,200,000
F	100,000	0	$18	6–12	11,111	$162	$1,800,000
TOTAL	227,400	>26,000 (>10,362)	∼$32 (average)	∼4.2 vials (average)	54,371	∼$133 (average)	$6,640,000

**Table 3:** 2010/11 sub-Saharan African antivenom output and market.

Company	Vials produced per year	Vials unsold in 2010	Wholesale cost per vial (US$)	Vials per average treatment	Complete treatments (average)	Cost of AVERAGE treatment	Value of African AV
A	12,000	0	$40	1–2 (mean 1.4)	8,500	$55	$480,000
B	2000†	0	$135	3–4 (mean 3.7); ≥25 LD50	<500	$500	$270,000
C	∼13,500	0	$80 (poly); $200 (mono)	6–10 (poly); 2 (mono)	∼1,600 (poly); ∼250 (mono)	$640 (poly); $400 (mono)	$1,140,000
E	150,000	0	$32	2–4 (mean 3)	50,000	$96	$4,800,000
F	200,000	0	$18 liquid; ($22 lyophilized)	6–12; >20–25 LD_50_	22,222	$162	$3,600,000
G	N/A			2–6 vials (mean 3.8)			
H	N/A (projected 20,000)			3–6 vials (mean 3.8)			
I	N/A						
TOTAL	**377,500**	0	∼$28∧	∼4.5 vials∧	83,072	∼$124∧	$10,290,000

(†based on 2007 company projections;∧ average;N/A = not yet available).

#### c. Antivenom quality

It is evident from product inserts and literature reviews that the potency of antivenom sold in sub-Saharan Africa varies widely. The average number of antivenom vials required to achieve effective neutralisation of a moderate envenoming, based on manufacturers' recommended doses, is 4.5 vials (range 1 to 12 vials). Doses for severe envenomings can be several times greater. Whilst proven effective antivenom products against African snake species do exist, it is highly concerning to note that recent peer-reviewed evaluations and published personal reports have indicated that two dominant products in the African market, which account for up to 90% of the total output, lack efficacy against some snake species to which they are targetted [2], [12], [33]–[35]. The actual number of effective antivenom treatments available in Africa, therefore, is potentially only a fraction of the 83,000 stated above, and may cover as little as 2.5% of the estimated need.

#### d. Antivenom cost

The wholesale cost of antivenoms for sub-Saharan Africa ranged from $18 to $200 per vial. The corresponding cost per *effective treatment*, using recommended doses, was $55 to $640, with an average cost of $124. Total company revenues from these products increased from $6.6 million in 2007 to approximately $10.3 million in 2010/11. The two largest manufacturers accounted for almost $8.4 million (81.5%) of revenues, despite recent concerns about the suitability of their products for use in some African markets.

#### e. Antivenom formulation

Of the 8 current and pending producers of sub-Saharan African antivenoms, 6 manufacture solely polyspecific products, one produces only monospecific, and one produces both polyspecific and monospecific antivenoms. One currently marketed and one future product consists of whole IgG antibodies purified with caprylic acid, while the remainder manufacture F(ab)′2 products. One company utilises ovine antisera instead of equine, and 6 offer lyophilised products.

### 3. Global antivenom market

#### a. Manufacturers

In 2007, 46 one-time antivenom manufacturers across 28 countries were surveyed and 35 reported current production of at least one type of snake antivenom for commercial, government or research purposes. Eleven organisations listed in various media as antivenom manufacturers either no longer produce snake antivenom or did not respond to the survey. Twenty-four of the 35 organisations producing antivenom operate on a commercial basis; 6 were purely government facilities manufacturing non-commercial antivenom for domestic purposes; and 5 companies did not provide financial data.

#### b. Antivenom output and capacity

Total global snake antivenom output by surveyed companies exceeded 4 million vials, although this equated to fewer than 600,000 effective treatments. This is well below the WHO's worldwide estimated requirement of at least 2 million treatments per year. Globally, twelve manufacturers reported having capacity to increase volume, which if realised could potentially double the current output.

#### c. Antivenom quality and formulation

As with the antivenoms in Africa, many commercially available antivenoms are associated with highly variable potency, ranging from 1 to >30 vials required to complete an effective treatment. A majority of products were produced using F(ab)′2, and only 3 manufacturers reported using Fab or intact IgG.

#### d. Antivenom cost

In 2007 wholesale prices for individual antivenoms across the global range of products ranged from $8 to $1338. The cost of treatment based on manufacturer recommended doses was calculated to be between $40 and $24,000. However case reports indicate that the number of vials required to successfully treat severe envenoming with some products may exceed the recommended amount [36], [37], with associated wholesale costs of over $35,000 per treatment [38] and even higher retail costs. Total company income from worldwide antivenom sales amounted to more than $60 million, and only two groups had annual antivenom sales exceeding $10 million.

**Figure 1:**
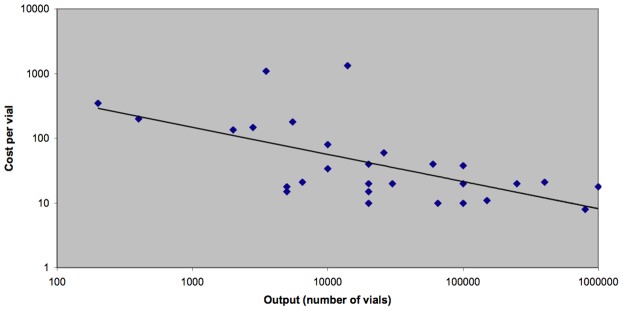
Antivenom price v output. Economies of scale mean that the cost per ampoule decreases as throughput increases.

There is a clear relationship between wholesale cost of antivenom and throughput (Figure 1), which has important implications for strategies seeking to increase the amount of antivenom produced globally. It was estimated by one company that costs could be reduced 5-fold from an 8-fold increase in output. Another company reported that doubling production would only increase costs by 10% and could potentially halve antivenom price. However, the retail price of antivenom is also heavily influenced by the market's ability to pay for it. On a per vial basis, antivenom developed for use in high-income countries is disproportionately more expensive, represented by the two out-lying plot points in Figure 1.

### 4. Attitudes to future antivenom production

All companies currently producing antivenom for sub-Saharn Africa indicated a willingness to increase output should market demand improve. Manufacturers identified factors that prevented them from raising production, despite a willingness to do so. Whilst not all manufacturers listed the same reasons, there was some concordance and the responses below have been listed in descending order of frequency:

- Lack of consistent market demand for antivenom products;
- Inconsistencies with manufacturers receiving payment.
- Corruption within some global markets and government agencies;
- Threats from black market re-sale of antivenom products;
- Lack of appropriate venom for immunogen preparation,
- A lack of certainty regarding appropriate distribution of their products;
- Inappropriate clinical use of antivenom products;
- Lack of adequate animals for raising antisera; and
- High costs of maintaining livestock for antivenom production;

## Discussion

This survey of antivenom manufacturers highlights the paucity of antivenom products for sub-Saharan Africa and the unhelpful variability that exists within the current industry. It also illustrates that despite the exodus of manufacturers in the 1970s and 1980s, willing producers do exist and they possess substantial unutilised production capacity. Unfortunately, inadequate government and non-government funding for procurement and regulatory oversight restrains production of commercial antivenom. This lack of investment is not only the reason for the current crisis in antivenom availability, but also represents the greatest challenge to future improvements in quantity and quality.

**Figure 2:**
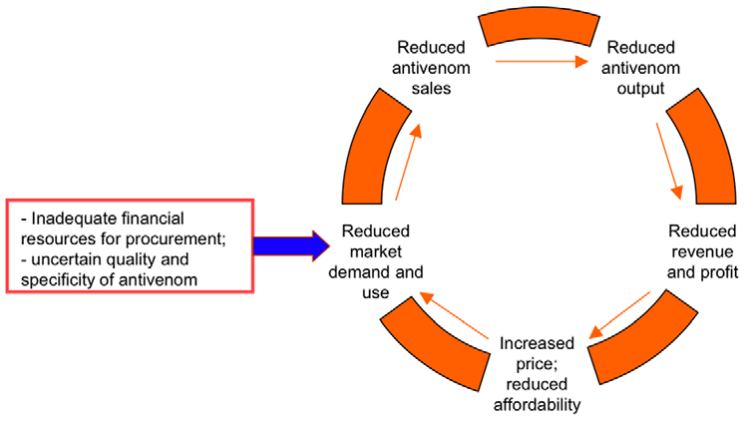
The self-perpetuating cycle responsible for the decline in antivenom production in sub-Saharan Africa. Inadequate financial support for antivenom production and variable quality have catalysed the collapse of the antivenom market, which is now characterised by deficient supply, deficient quality control, rising prices and poor profitability.

Although inexpensive and efficacious antivenoms do exist, and compelling moral and legal arguments advocate increased purchase and distribution [39], [40], a lack of funding for antivenom acquisition and regulation of quality standards has catalysed the vicious cycle responsible for the decline in production and use over the last 30 years (figure 2). This cycle has also contributed to conditions that have allowed lesser quality products and inappropriate marketing to emerge. The arrival of new manufacturers and the presence of spare capacity within some current facilities provide hope, but uncertain market conditions and inadequate financial support will continue to restrict growth of trustworthy antivenoms.

This cycle is a variation on that proposed by Stock et al in 2007 [9], and demonstrates the importance of future financial stimulus in reinvigorating competition and viability of the antivenom market. Inadequate financing within the antivenom industry is the major factor underpinning its decline over the last 40 years, and strategies to solve this crisis must recognise and unwind the economic and commercial drivers on both sides of the supply and demand equation. It is unrealistic to expect that pharmaceutical companies will commit to long-term production of antivenom for an inconsistent and unreliable market that is starved of investment. Even if greater volumes of appropriate antivenom could be produced, without adequate subsidisation it will be priced out of range for most snakebite victims living in underprivileged rural and remote areas. Similarly, corporate executives and regulatory bodies must also accept that there exists a moral imperative for them to contribute their expertise and capabilities, and that existing business models and production frameworks may be inappropriate for the supply of humanitarian products to developing countries.

Encouragingly, there has been a small increase in financial support for the development and procurement of new African antivenoms between 2007 and 2010. Whilst the >$60 million in global antivenom revenue and $10.3 million from African antivenom sales are small by pharmaceutical standards, this represents valuable investment and an encouraging base from which the industry can grow. Better utilisation of spare production capacity and improved economies of scale will produce greater yields, reduce costs, increase revenues and further enhance the commercial viability of antivenoms.

The second major problem eroding the antivenom market is the lack of accountability in quality standards. Possessing the capacity to produce vast amounts of antivenom for sub-Saharan African communities is meaningless if the products are poorly made and ineffective against the snakes in those regions. A current lack of interest, insufficient investment and poor competition are allowing unscrupulous behaviours within the marketplace to go unchecked. Given the ongoing severe shortage of antivenom and the continuing high incidence of envenoming, it is not surprising that opportunistic manufacturers seek to fill the void. The advent of seemingly inexpensive, but low quality or inappropriate antivenoms with poor neutralising ability, not only compromises the reputation of antivenoms in general but also drains important financial resources away from proven snakebite treatment programs and products. Some manufacturers have cited this uneven playing field as a key impediment to future innovation and productivity. Nevertheless, the very high volume output by some manufacturers of alleged inappropriate products still make them key players in the antivenom industry, and potentially integral to future strategies for increasing output of higher quality products. Improving standards and maximising efficiencies ought to be the common goal for all manufacturers.

The three groups with emerging new African antivenoms provide hope for the future [41]–[44], however ensuring that these products, as well as existing antivenoms, are of sufficient quality to be incorporated into a properly funded and sustainable market is paramount [8]. The final quality control checkpoint for all antivenoms entering a country should be the national regulatory authorities. It is essential that NRAs are adequately resourced and transparent to ensure the integrity and robustness of their mechanisms are above reproach. Linking funds for antivenom procurement to improved quality control and assurance measures would enhance the crucial role of local regulatory bodies and incentivise the maintenance of minimum standards.

Antivenom's usually rapid and curative effects make it a highly cost-effective intervention [40], and together with snakebite's surpassing morbidity and mortality [6], ought to attract attention from global health funding bodies. If improved efficiencies, technical support and collaboration within the antivenom industry were achieved, the cost of an effective antivenom treatment would fall below the current average of $124, and may ultimately be significantly less than $100. Supplying sufficient quantities of antivenom to the whole of Africa at that price would require an annual input of less than $30–$50 million, which is considerably lower than the budgets for many other global health programs. Leadership and support from groups such as the Global Snakebite Initiative and the World Health Organisation may help to secure essential funds from donors and provide important coordination, transparency and accountability. It will also help to recruit and reform manufacturers capable of contributing a greater supply of effective and appropriate antivenoms.

The declining availability of high quality antivenom in sub-Saharan Africa is a real and unnecessary tragedy, and constitutes a major neglected global health concern. The amount of suitable antivenom marketed in these countries has fallen to crisis levels, representing only a fraction of the amount required. Although recent output of antivenom for Africa has increased, and the number of manufacturers able to boost production is growing, inadequate financial support and market uncertainty continue to suppress growth and compromise quality standards. The provision of sufficient funds to identify satisfactory antivenoms, maintain quality control, maximise efficiencies and increase procurement is desperately needed to break the vicious cycle that currently constrains the antivenom industry. The mechanisms to achieve this are realistic and available; science, business and government must collaborate to secure a brighter future for snakebite victims in developing countries. Only then will the goal of providing effective, safe and affordable antivenoms to all who need them, be realised.
